# Cancer Stem Cell Hierarchy in Glioblastoma Multiforme

**DOI:** 10.3389/fsurg.2016.00021

**Published:** 2016-04-15

**Authors:** Amy Bradshaw, Agadha Wickremsekera, Swee T. Tan, Lifeng Peng, Paul F. Davis, Tinte Itinteang

**Affiliations:** ^1^Gillies McIndoe Research Institute, Wellington, New Zealand; ^2^Department of Neurosurgery, Wellington Regional Hospital, Wellington, New Zealand; ^3^Centre for Biodiscovery, School of Biological Sciences, Victoria University of Wellington, Wellington, New Zealand

**Keywords:** glioblastoma multiforme, cancer, cancer stem cell, markers, hierarchy

## Abstract

Glioblastoma multiforme (GBM), an aggressive tumor that typically exhibits treatment failure with high mortality rates, is associated with the presence of cancer stem cells (CSCs) within the tumor. CSCs possess the ability for perpetual self-renewal and proliferation, producing downstream progenitor cells that drive tumor growth. Studies of many cancer types have identified CSCs using specific markers, but it is still unclear as to where in the stem cell hierarchy these markers fall. This is compounded further by the presence of multiple GBM and glioblastoma cancer stem cell subtypes, making investigation and establishment of a universal treatment difficult. This review examines the current knowledge on the CSC markers SALL4, OCT-4, SOX2, STAT3, NANOG, c-Myc, KLF4, CD133, CD44, nestin, and glial fibrillary acidic protein, specifically focusing on their use and validity in GBM research and how they may be utilized for investigations into GBM’s cancer biology.

## Introduction

**Figure 1:**
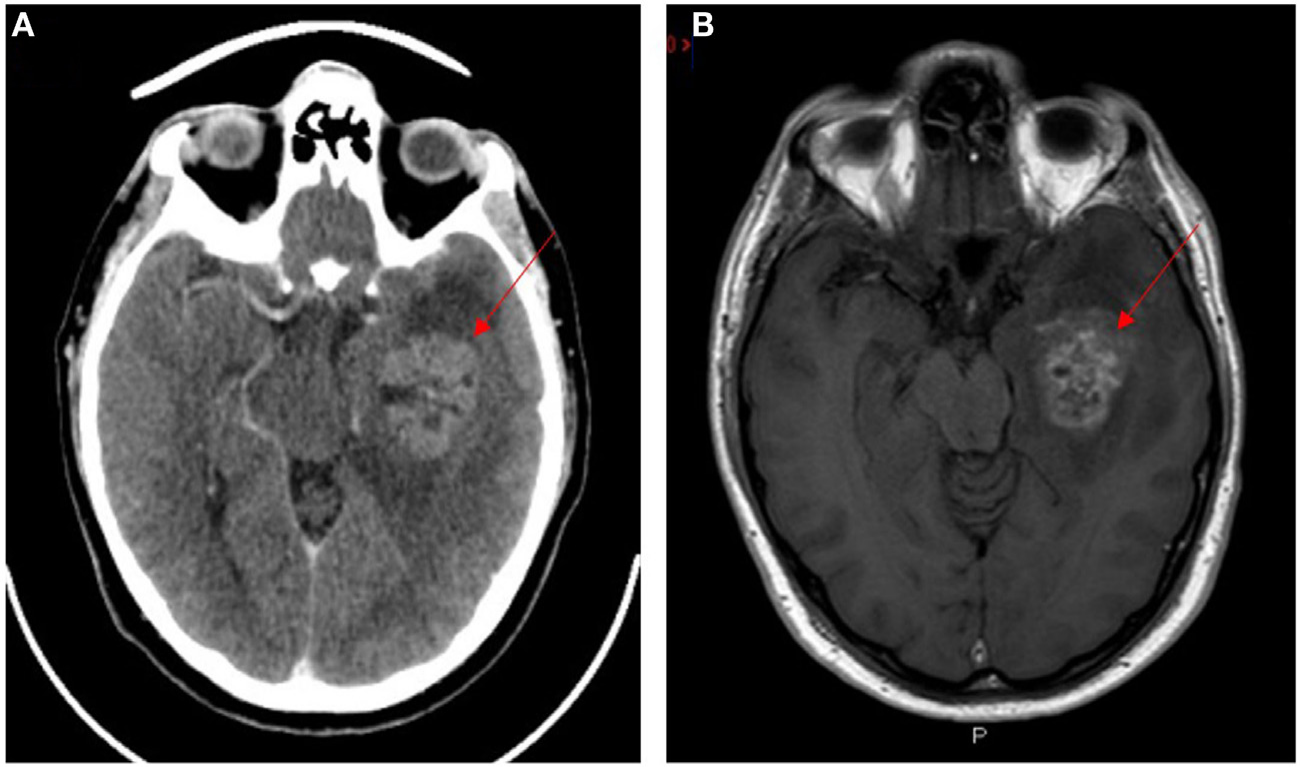
**CT (A) and T1 weighted MRI (B) scans with contrast of a patient with glioblastoma multiforme**. Lesion indicated by red arrows.

Glioblastoma multiforme (GBM), a grade 4 astrocytoma, is the most aggressive form of glioma (1, 2) with the median survival of approximately 25 months following treatment (3). Despite advances in cancer research and treatment over several decades, there has only been a 2% improvement in 5-year survival (4). GBM has been shown to be resistant to radiotherapy and chemotherapy (5–7) and invariably recurs following surgical resection (8) and chemoradiation (9). GBM typically shows a space-occupying lesion with heterogeneous rim enhancement, causing mass effect with surrounding edema on computerized tomography (Figure 1A) and magnetic resonance imaging (Figure 1B).

**Figure 2:**
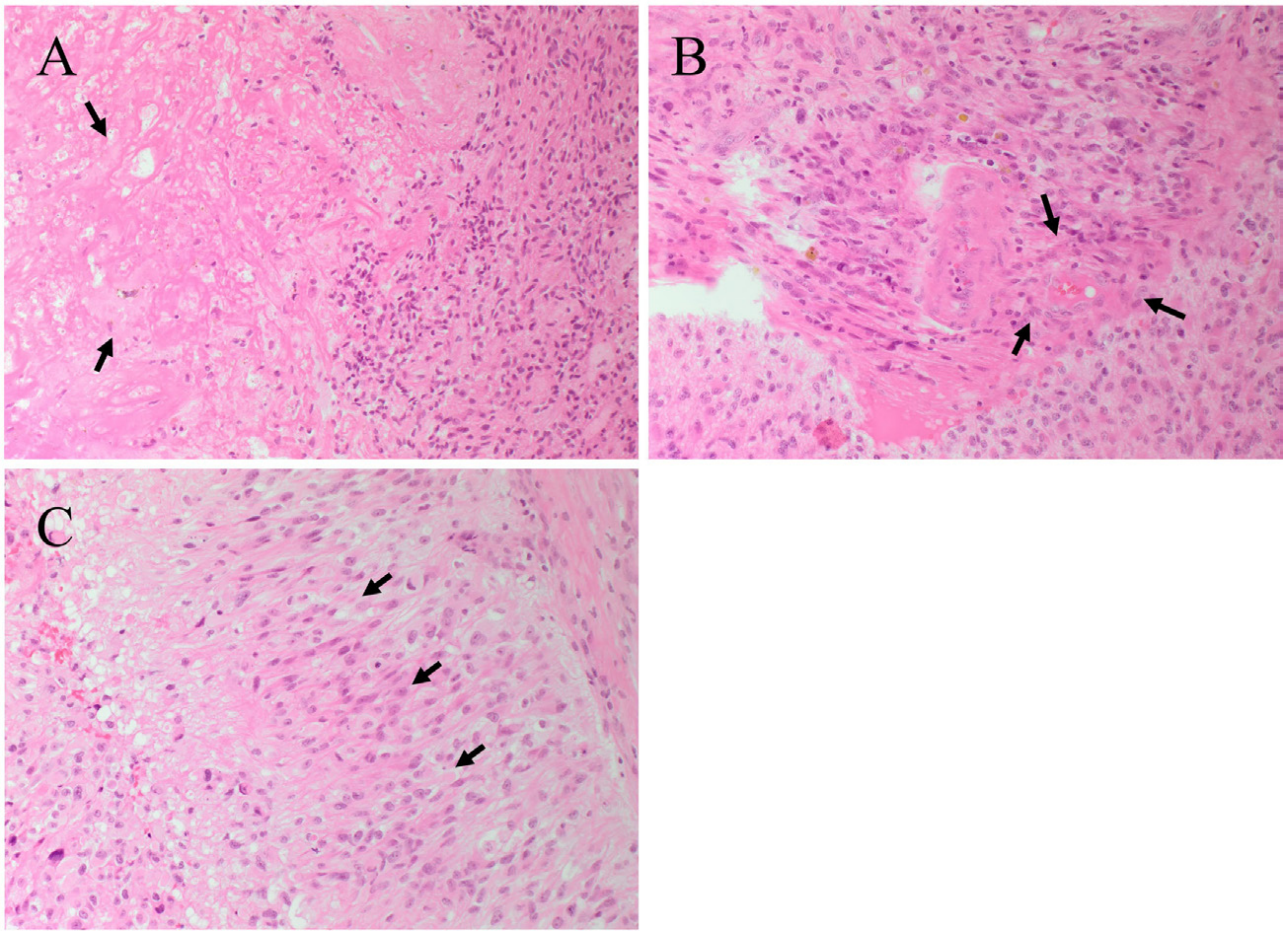
**Hematoxylin and eosin staining of a glioblastoma multiforme**. **(A)** The interface between tumor cells and the area of necrosis. The necrotic area (*arrows*) show greatly reduced nuclear staining. **(B)** Proliferation of the endothelial cells (arrows) within a microvessel. **(C)** Palisading cells (*arrows*) around the necrotic area. Original magnification: 200×.

The presence of central necrosis (Figure 2A, arrows) and marginal proliferation of endothelial cells (microvascular hyperplasia) (Figure 2B, arrows) are hallmark histological features that separate GBM from lower grade glial tumors. Another characteristic feature of GBM is the presence of palisading cells around the area of necrosis (Figure 2C, arrows), which is widely regarded as a poor prognostic hallmark of GBM (10, 11). Increased mitosis, hypercellularity, atypical nuclei and cellular pleomorphism, and the development of lumina, reminiscent of kidney glomeruli (10, 12), are other histological features of GBM. Combinations of some or all of these features result in marked histological heterogeneity, indicating that GBM tumors can change and grow rapidly even while the central bulk of the tumor undergoes necrosis (13). While much is known about the histological features and chromosomal abnormalities (14, 15) in GBM, the molecular characteristics and the origin of the lesion are not fully elucidated.

This article reviews the data on cancer stem cell (CSC) markers currently used in GBM research and attempt to place them in the context of a hierarchical model of cancer.

## Models of Cancer

**Figure 3:**
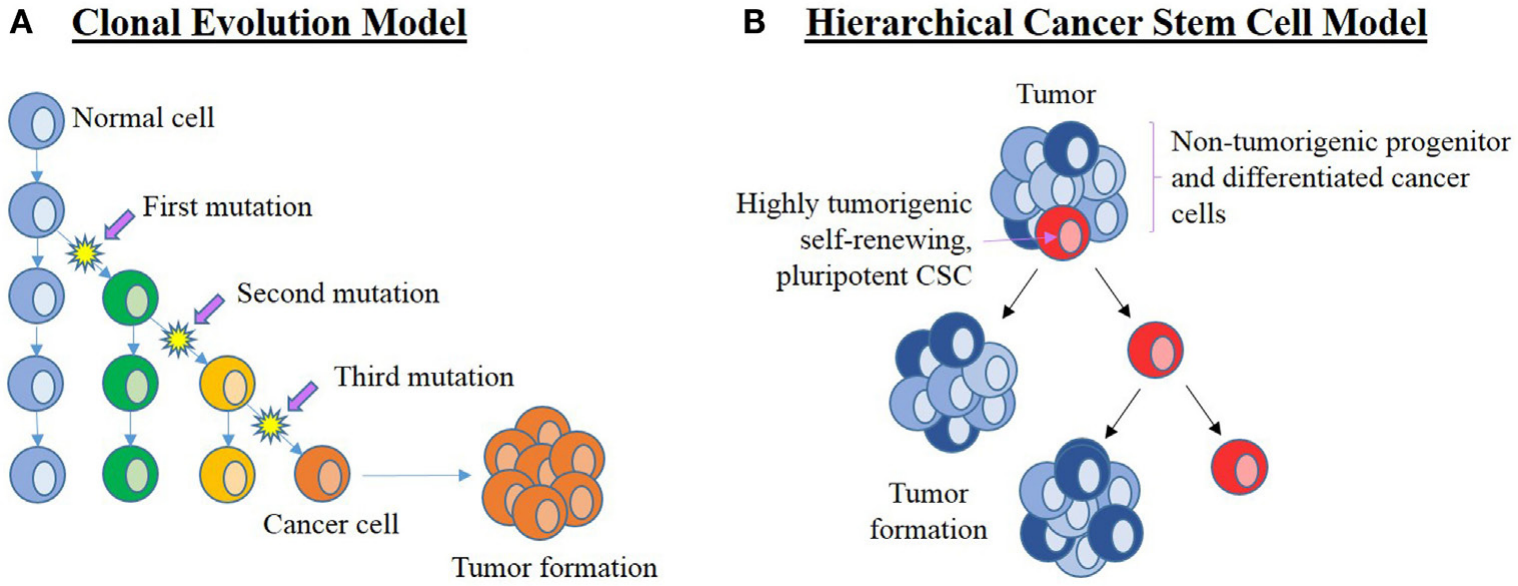
**Current leading models of carcinogenesis**. **(A)** The clonal evolution model hypothesizes that a normal cell (blue) within the organism undergoes a series of mutations to form a cancer cell (orange) that clonally expands and form the bulk of the tumor. Successful treatment must, therefore, eliminate all cancer cells. **(B)** The cancer stem cell (CSC) hierarchical model proposes that the origin of cancer being CSCs (red) that are pluripotent and self-renewing. They are highly tumorigenic with the ability to establish new tumors. CSCs divide asymmetrically to form new CSCs and progenitor (dark blue) cells that in turn give rise to differentiated cancer cells (light blue) that form the bulk of the tumor. These downstream cancer cells are low or non-tumorigenic. Adapted from Adams and Strasser (16).

The two current concepts on the origin of cancer and its continued propagation are: (1) the clonal evolution (or stochastic) model (Figure 3A) (16, 17), and (2) the hierarchical CSC model (Figure 3B) (16, 18).

The clonal evolution model of cancer proposes cumulative genetic mutations that occur over time in a normal cell, leading to the formation of a cancer cell that clonally expands to form identical copies, each with identical tumorigenic potential (16, 19). If these changes confer a selective advantage to a particular cell, then this allows the selected “clone” to outcompete other potential tumor forming clones (17). Propagation of this selected clone means that a substantial number of cells in the tumor are able to maintain tumor growth, so any effective treatment would require the elimination of all clonal cells, a theory that is inconsistent with the identification of CSCs in cancer (16).

**Figure 4:**
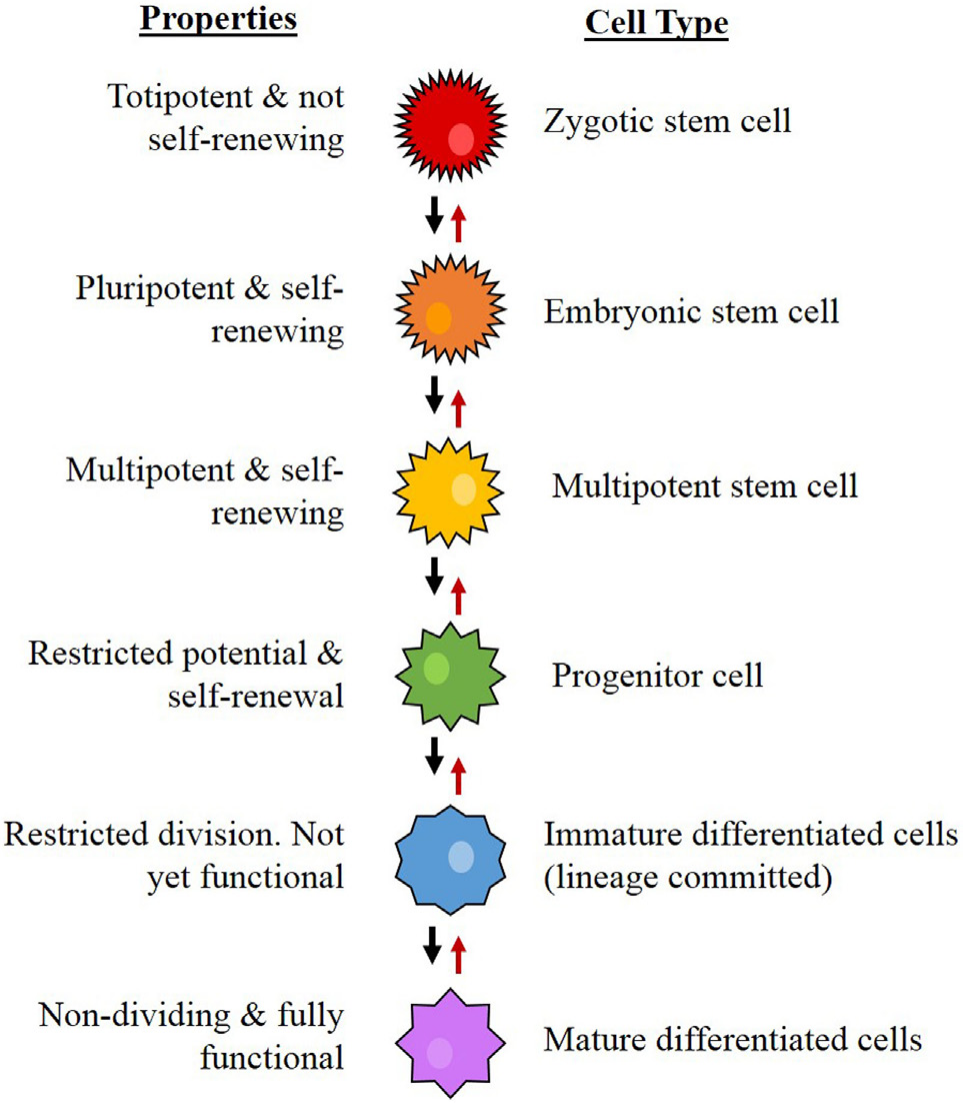
**The proposed hierarchy for neural stem cell differentiation**. The system begins with the most primitive and multipotent cell and moves through stages of differentiation to the most restricted cell. Concept from Gage (24).

Stem cells are cells that possess the capacity for self-renewal, proliferation, and differentiation (20–22). From a hierarchical viewpoint, embryonic stem cells (ESCs) are the most primitive cells within a biological system, and are considered pluripotent in that they are capable of differentiating into any type of cell in a particular organism (23). Downstream from ESCs, which are progenitor cells, a group that includes neural stem cells (NSCs) (24), mesenchymal (MES) stem cells (25), endothelial progenitor cells (26), and hematopoietic stem cells (HSCs) (27). These cells are multipotent, have more restricted lineage differentiation capacity, and, therefore, are no longer pluripotent (24). From here, the multipotent NSCs further differentiate, giving rise to more downstream progenitor cells with reducing differentiation, mitotic, and self-renewal potential, ultimately forming the majority of the organism (Figure 4) (24, 28).

Current literature uses the terms *stem cell* and *progenitor cell* interchangeably, essentially lumping these two cell types together (29–34). However, the validity of this practice has been questioned (35) as progenitor cells and stem cells differ in terms of hierarchy and biology and, therefore, should be regarded as distinct entities. Stem cells are multipotent with an unlimited capacity for self-renewal, whereas progenitor cells are most often unipotent with restricted capacity for self-renewal. Distinguishing between stem cells and progenitor cells in cancer is important in the understanding of the CSC concept for carcinogenesis. However, as they presumably belong to a spectral continuum distinguishing between the two populations remains a challenge.

The hierarchical CSC model of cancer proposes that a tumor arises from CSCs generated by mutations in either normal ESCs or progenitor cells, which may be present at birth or accumulated over time resulting in cells possessing the ability for uncontrolled growth and propagation (36–39). Recent studies have also observed the ability of non-CSCs to “de-differentiate” into CSCs due to epigenetic or environmental factors, which further increases the complexity of tumor biology and treatment (40). Cancer consists of a heterogeneous population of cells, proposed to arise from CSCs. Cells in a tumor are thought to be structured in a similar hierarchical manner to normal tissues, ranging from the most primitive cells to the most mature cells (Figure 4) (24, 41). Within a tumor, there may only be a small number of CSCs that are highly tumorigenic (Figure 3B) (16) and have the capacity to divide asymmetrically giving rise (1) to additional CSCs that migrate to form new tumors and (2) to downstream progenitor cells and differentiated cancer cells that possess no or low tumorigenic potential (42) and form the main bulk of the tumor (38, 41, 43).

It is important to note that these two different hypotheses may not be mutually exclusive, as clonal evolution has been shown to play a role in the formation of CSCs (44, 45).

## CSCs in Glioblastoma

A combination of clinical evaluation and genome-wide expression profiling has revealed that high-grade gliomas can be separated into four subtypes: proneural (PN), MES, neural, and proliferative (or classical) (15, 46). There remains some debate regarding the number and defining characteristics of these subtypes (46), but some criteria, such as chromosomal deletions and molecular markers (such as Notch and VEGF) have been proposed (47). The existence of multiple subtypes provides another explanation for therapy resistance in GBM, which needs to be taken into account when characterizing GBM cells (7). This adds another level of complexity to the study of GBM, as in addition to the known intra-tumoral cellular heterogeneity, there is also a degree of inter-tumor cellular heterogeneity.

In addition to the tumor subtypes, CSCs isolated from high-grade gliomas are also categorized into two distinct groups: PN and MES (48, 49). Several studies have adopted the term glioma stem cells to describe CSCs found in GBM (40, 49, 50), but for the purpose of clearly differentiating between stem cells in lower grade gliomas and those found in GBM, this review will use the term glioblastoma cancer stem cells (GBCSCs). GBCSCs are thought to originate from either neuronal stem cells or de-differentiate from normal brain cells, such as astrocytes and oligodendrocytes (18, 40), although this de-differentiation is not universally accepted (46). PN GBCSCs appear to share similarities with fetal NSCs, while MES GBCSCs more closely resemble adult NSCs (46, 51). MES GBCSCs are more aggressive, invasive, angiogenic, and resistant to radiotherapy than PN GBCSCs. MES GBCSCs are predominantly derived from primary GBMs that arise *de novo*, whereas PN GBCSCs reside in both Grade III gliomas and GBM (49, 52). Primary GBM can also contain multiple (polygenomic) or single (monogenomic) tumor cell clones and different genetic clones impact on tumorigenesis differently (53). However, even genetically diverse clones possess the stem cell markers CD133, CD15, A2B5, and CD44 (53), which suggests that despite the large amount of inter- and intra-tumor cell heterogeneity and the influence of the brain tumor microenvironment (54), at least some (if not all) stem cell markers remain consistent, thereby providing arguably one of the best targets for cancer therapy.

## Molecular Markers in CSCs and GBCSCs

ESCs were originally identified and characterized from cells of the inner cell mass (ICM) in an embryonic blastocyst (55–57). ESCs and their more differentiated progeny all express a variety of markers, ranging from surface markers to transcription factors (57). As cells with stem cell properties isolated from cancers have been proposed to originate from ESCs, they also express these markers, making it possible to both identify and isolate CSCs using these markers (57, 58). Schoenhals et al. (58) showed that at least one of the ESC markers OCT-4, SOX2, KLF4, and c-Myc was expressed in 18 out of 40 cancer types investigated. Takahashi et al. (59) obtained induced pluripotent stem cells (iPSC) by transduction of these same markers into murine embryonic fibroblasts. iPSCs are made by reprograming adult somatic cells via transfection with specific markers, causing them to de-differentiate and regain ESC-like characteristics (60). Many more studies using additional ESC markers have resulted in a growing body of evidence for the presence of CSCs in many cancer types (61, 62). CSCs have been identified in lung (63), breast (64), head and neck (65), prostate (66, 67), pancreatic (68, 69), and colon (70, 71) cancers. GBCSCs were first identified by Ignatova et al. (72) and their presence has been confirmed in several other studies (20, 73–77). The list of proposed GBCSC markers includes CD133, nestin, NANOG, SALL4, STAT3, SOX2, c-Myc, Olig2, Bmi1, CD44, L1CAM, and KLF4 (1, 78–81).

**Figure 5:**
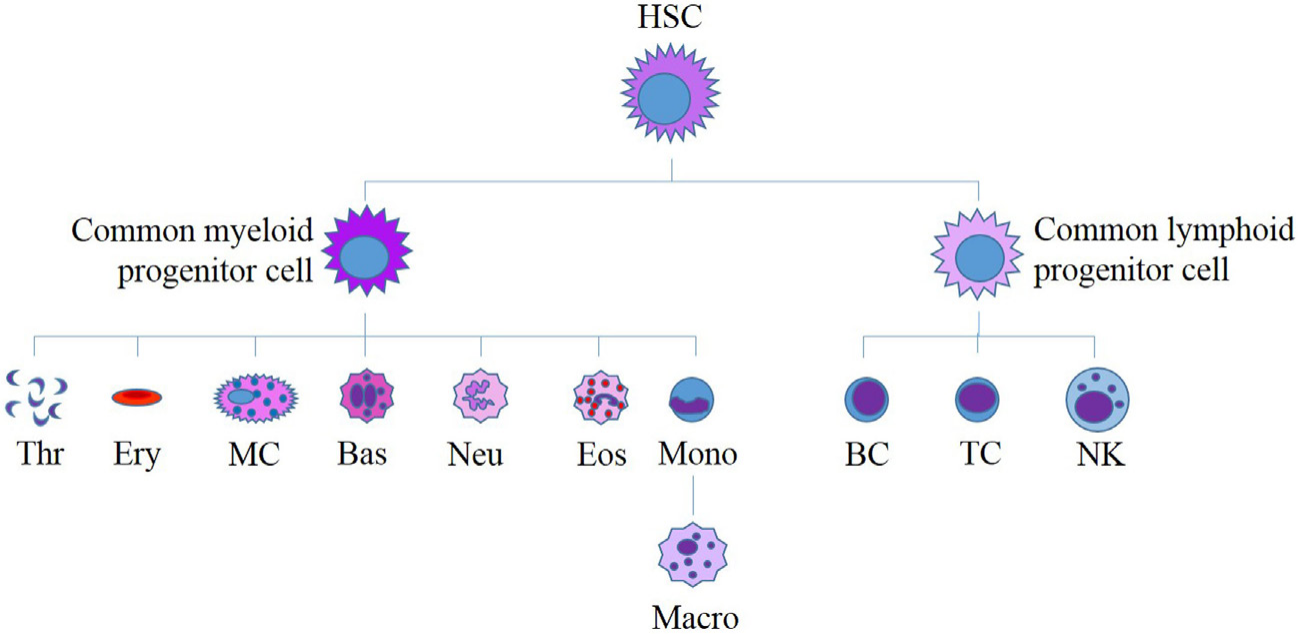
**Current model for human hematopoiesis**. All myeloid and lymphoid cells originate from a single hematopoietic stem cell (HSC). HSCs differentiate to form myeloid and lymphoid progenitor cells, which in turn differentiate to produce all of the diverse cells found in human blood. HSC, hematopoietic stem cell; Thr, thrombocytes; Ery, erythrocytes; MC, mast cell; Bas, basophil; Neu, neutrophil; Eos, eosinophil; Mono, monocyte; Macro, macrophage; B, B-cell; T, T-cell, NK, natural killer cell. Adapted from Bartis and Pongracz (86).

The literature on normal ESCs describes a hierarchical differential expression pattern of stem cell markers, with ESC markers at the top of the hierarchy and progenitor cell markers more downstream (20, 28, 82–84). This is consistent with the observation that during formation of blood cells, HSCs rarely divide (85), making them slow in responding to any environmental changes and that they produce slightly more differentiated progenitor cells (Figure 5) (86). These cells are more numerous and proliferative, making them much more adaptable to change but still able to drive blood cell formation (87). Evidence for core stem cells and more malleable progenitor cells has also been found in some forms of cancer (38, 88, 89). In GBM, despite the known cellular heterogeneity present both within tumors and between patients (40, 49, 53) and the discovery of GBCSCs (52), characterization studies of the stem cell markers present on different GBCSC subtypes or at different tumor stages are relatively rare. The hierarchical model of cancer which proposes that a core group of stem cells exists at the top of the tumor hierarchy, from which other more differentiated cells are formed, descending from the most primitive cells to the most mature cells that make up the bulk of the tumor mass, remains relatively unexplored in GBM.

This review aimed to provide a perspective on CSC markers SALL4, OCT-4, SOX2, STAT3, NANOG, c-Myc, KLF4, CD133, CD44, nestin, and Glial fibrillary acidic protein (GFAP) reported in GBM, and attempt to place these markers in the context of the GBM CSC hierarchy, from the most primitive ESC markers to the more mature. These markers are presented in two categories: embryonic CSC markers and neural progenitor CSC markers.

## Embryonic CSC Markers

### SALL4

SALL4 is a spalt-like C2H2 zinc-finger transcription factor that is expressed on ESCs in a similar manner to OCT-4 and SOX2 (90, 91). SALL4 is mandatory for the development of the ICM to ensure zygotic survival and maintenance of ESC pluripotency (90, 92, 93). Interaction between SALL4 and NANOG has also been confirmed by co-immunoprecipitation experiments and it has been suggested that they work together in a similar pairwise manner to ESC markers OCT-4 and SOX2 to regulate transcription (94). However, other evidence suggests that, as well as NANOG, this regulatory function also has a greater involvement of OCT-4, SOX2, c-Myc, and KLF4. Yang et al. (91) report that when the level of the SALL4 protein is reduced, the levels of all four of the aforementioned ESC proteins also decrease, suggesting a relatively diverse role for SALL4. SALL4 plays a role in multiple types of cancers (95–97) and has been previously used as a CSC marker. It has also been demonstrated that SALL4 is expressed at a higher level in gliomas than in normal brain tissue and that increased levels correlate with a poor prognosis (80). Additionally, inhibition of SALL4 reduces cellular proliferation in gliomas and stimulates apoptosis (98). Di Tomaso et al. (99) find that CSCs in GBM express SALL4 and that these same cells also express NANOG. However, the use of SALL4 as a marker for CSCs in GBM is limited to a small number of reports (80, 98, 100, 101).

### OCT-4

Together with NANOG, the transcription factor, OCT-4, is required for propagation of ESCs and they both work synergistically with SOX2 to achieve this regulation (102). OCT-4 is essential for pluripotency and mammalian embryonic development (103). It has also been associated with cancer, functioning as a driver for the self-renewal of CSCs (78, 104). OCT-4 is expressed by glioma cells, but not normal brain tissue, and is implicated in the pathogenesis of GBM (105, 106). Indeed, OCT-4 together with SOX2 and NANOG is expressed in most if not all gliomas and their expression correlates with tumor aggressiveness with GBM cells showing greater nuclear staining for OCT-4 and SOX2 (104). Furthermore, most cells expressing OCT-4 also express SOX2 and NANOG (104). Hence, OCT-4, SOX2, and NANOG are thought to be key players in the transcriptional regulation of CSCs.

### SOX2

SOX2 is a member of the family of transcriptional co-factors that are associated with various developmental milestones and is over-expressed in tumors (107, 108). It plays a role in maintaining pluripotency in several types of cancer, including rectal (109), breast (110), and lung (111) cancers. SOX2 is also over-expressed in GBM with little detected in normal brain tissue (1). Additionally, GBM demonstrates greater SOX2 mRNA expression than lower grade tumors (104). Along with OCT-4 and NANOG, SOX2 has been used in numerous studies to characterize iPSCs derived from somatic cells (59, 112, 113), demonstrating that SOX2 is critical for stem cell maintenance. Furthermore, SOX2 inhibition using shRNA halts tumor growth when GBM cells are transplanted into immunodeficient mice (114).

Although SOX2 has been implicated as a transcriptional regulator, it has also been proposed as a neural progenitor cell marker. Expression of SOX2 is essential for cells from the neural tube in chicks to maintain progenitor characteristics (115). There is also a body of evidence on co-expression of SOX2 and nestin and as such SOX2 has been used in some studies as a progenitor cell marker (116–119). Ellis et al. (120) show SOX2 as a persistent NSC marker that is present throughout the entire development of the mouse. The expression of SOX2, therefore, appears to be maintained even after stem cells have progressed through different stages of differentiation.

### pSTAT3

Signal transducers and activators of transcription (STAT) proteins are both activated by cytokines and regulate many cytokine and growth factor responses (121). STAT3 has more generalized functions than the rest of the STAT family and has been implicated in cell-cycle signaling, cell survival, and ESC self-renewal and pluripotency (122–124). The latter activity has been proposed to be maintained via the leukemia inhibitory factor (LIF) pathway, in which LIF binds to its receptor and produces phosphorylation of STAT3 that subsequently translocates to the nucleus, triggering the expression of other ESC-associated proteins, such as KLF4, SOX2, SALL4, and c-Myc (62, 125, 126). Loss of STAT3 expression has been shown to reduce ESC self-renewal, but enhance cellular differentiation, resulting in embryo lethality in mice (127). This would, therefore, indicate that ESC expression of STAT3 is essential, but that it also performs multiple other functions in adult tissues, such as cytokine release and cell signaling (124), suggesting STAT3 expression persists on further differentiated cells and, therefore, cannot be used purely as a primitive ESC marker.

Abnormal STAT3 signaling has also been associated with promoting cellular proliferation, weakening the immune system, and promoting angiogenesis and inflammation in cancer (123, 128). There is ample evidence for the role of STAT3 in cancer, with its activation contributing to cancers in the head and neck (129), breast (130), prostate (131), thyroid (132), skin (melanoma) (133), and GBM (134–136). In comparison to normal brain tissue and cells, particularly astrocytes, GBM expresses high levels of STAT3 and inhibition of this molecule results in the induction of apoptosis and cessation of tumor proliferation (81). Multiple subsequent studies of STAT3 in GBM have demonstrated its downregulation or inhibition leads to reduced tumor growth, suggesting a potential target for cancer treatment (137–141). However, as STAT3 is also required for non-cancer cell function, any form of inhibition will not be specific to the tumor and will likely result in major side effects for the patient (7, 142).

### NANOG

NANOG is an ESC transcription factor and its expression has been associated with multiple types of cancer, including those affecting the lung (143), oral cavity (144), breast (145, 146), and prostate (147). It has also been implicated in the regulation of GBM and has been found to be highly expressed in stem cells extracted from the cerebellum and medulloblastoma (104, 148–150). NANOG modulates GBM stem cell tumorigenicity, clonogenicity, and proliferation (151). Inhibition of NANOG in GBM prevents tumor proliferation and invasion (152). It is proposed that together with OCT-4 and SOX2, NANOG is responsible for ESCs’ capacity to maintain their pluripotency and self-renewal (7, 153). Deletion of NANOG from murine ESCs results in a loss of pluripotency (154) and NANOG has been used as a marker in the induction of pluripotent stem cell characteristics in normal human fibroblasts (112, 155). Current data implicate a role for NANOG in the regulation GBCSCs.

### c-Myc

c-Myc is a member of the family of *Myc* genes, although only c-Myc, l-Myc, and N-Myc have been linked to tumor growth, and as such they have been termed nuclear oncogenes (156, 157). Upregulated c-Myc has been linked to cellular proliferation (158, 159). The deletion of c-Myc from rat fibroblast lines resulted in a prolonged cellular doubling time (160) and proved fatal to murine embryos, indicating its importance in embryonic development (161). Furthermore, c-Myc can be used to induce cellular de-differentiation, resulting in iPSCs (112).

c-Myc has been implicated in the pathogenesis of lung (162), pancreatic (163), prostate (164), and breast (165, 166) cancers as well as medulloblastoma (167) and GBM (168). Despite its experimental use in generating iPSCs, there is evidence indicating that c-Myc may be more of a marker for progenitor cells rather than ESCs. Successful generation of iPSCs without the expression of c-Myc implies that the oncogene is not essential for cellular de-differentiation (169). Additionally, in normal lung tissue c-Myc expression is strongest in hyperplastic alveolar type II pneumocytes, also known as bronchopulmonary progenitor cells (170). c-Myc also enhances the tumor forming capacity of nestin-expressing progenitor cells in medulloblastoma (171). This would suggest that c-Myc is expressed on progenitor cells, although its role as a neural progenitor cell marker is not fully established. Despite this, c-Myc has been strongly associated with GBM, CSC maintenance, and self-renewal, and its over-expression has been correlated with the poor prognosis of GBM (168, 171–173).

### Krüppel-Like Factor 4

Krüppel-like factor 4 (KLF4) is a transcription factor involved in cell proliferation, differentiation, and apoptosis (174). It is a member of the KLF family characterized by the presence of Cys2/His2 zinc fingers (57, 175). KLF4 is essential for the maintenance of pluripotency and self-renewal of ESCs (176, 177) and is one of the factors, along with OCT-4 and SOX2, required to re-program fibroblasts to generate iPSCs (59, 112, 169). It is, therefore, not surprising that KLF4 over-expression is associated with cancer (58, 178). KLF4 was first identified as a potential oncogene in 1999 (179) and since then its over-expression has been shown to induce cellular dysplasia, similar to that found in squamous cell carcinomas (180). More recently, it has been shown that KLF4 is over-expressed in 70% of breast cancer specimens (178). However, there is growing evidence indicating that KLF4 actually inhibits tumor formation and metastasis in many types of cancer (181–185).

A possible explanation for these discrepancies has been proposed, suggesting that the cell-cycle inhibitor p21 can act as a “switch” between suppression and proliferation (186). It is hypothesized that KLF4 can activate p21-induced cell-cycle arrest and prevent tumor proliferation, but can also inhibit p53, blocking both cell senescence and apoptosis. These responses are also thought to be influenced by the cellular context. For example, it has been theorized that inhibition of p21 by additional pathways such as Ras or the adenoviral oncoprotein E1A can override the activation signals of KLF4. Therefore, inhibition of both apoptosis via p53 and cell-cycle arrest via p21 induces tumor formation. KLF4 expression in the first scenario can produce completely opposite outcomes for different pathways, yet in the second produces only one outcome no matter which pathway is activated. This observation may explain the aforementioned contrasting results for KLF4 and has been supported by further studies in the area of cell-cycle regulation (187–189), although the exact mechanism of “switching” remains unclear. Unfortunately, the apparent heavy reliance of KLF4 function on other proteins and inconsistencies in its expression make it difficult to use KLF4 as a CSC marker.

Information on KLF4 expression in GBM is limited. An analysis of gene expression data indicates that KLF4 is over-expressed in brain tumors, with no specific data on GBM (58). A more recent study shows that micro-RNA targeting of KLF4 suppresses tumor growth in GBM cells (190) but the role of KLF4 in GBM remains undetermined.

## Neural Progenitor CSC Markers

### Nestin

The nestin gene (previously known as Rat. 401), the neuroepithelial stem cell gene, encodes a novel intermediate filament that does not fit into one of the five classes of intermediate filaments that have already been defined (191). Nestin is expressed in several types of cancer (191–193) and it is strongly associated with GBM (20, 21, 73, 194–196). Increased nestin expression has been associated with higher grade gliomas and lower patient survival rates (197). Additionally, inducing differentiation of GBM cells leads to downregulation of nestin (198). It also binds to a large percentage of cells in the mammalian embryonic brain and its presence is correlated with cellular propagation during the development of the central nervous system (199, 200). These data, plus the observation that nestin-expressing cells have the ability to differentiate into multiple cell types (72), implicate nestin as an effective stem cell marker. However, existing evidence indicates that nestin is more of a neural progenitor cell marker as it is found on immediate neuron precursor cells (199, 201) and is downregulated when precursor cells differentiate into glial cells or neurons (201). Nestin is currently used as a marker for cells immediately preceding the dedication of CNS cells to a restricted lineage (202).

### Glial Fibrillary Acidic Protein

Glial fibrillary acidic protein is an astrocyte maturation marker commonly used as a histological marker for tumors of glial origin known to be involved in normal astrocyte functions (203, 204). GFAP has been used previously to identify differentiated cells (20, 73), but some evidence indicates that astrocytes found in the subventricular zone (SVZ) of the mammalian adult brain are actually NSCs and are precursors to neurons (205). NSCs from the postnatal and adult brain have been found to express GFAP, but not NSCs from the early embryonic brain (206), indicating that GFAP is a marker of more mature glial cells. This would, therefore, suggest that GFAP is a progenitor rather than an ESC marker. GFAP has been previously shown to be co-expressed with nestin in GBM cells (207) and is over-expressed in the serum and peripheral blood of GBM patients in comparison to healthy controls (208, 209). However, the proportion of GBM patients with GFAP positivity varied greatly between these two studies. The study on serum found GFAP over-expression in 80% of GBM cases (208) [a finding that has been replicated recently (210)], whereas the peripheral blood study found that GFAP was over-expressed in only 20.6% of patients. It is possible that this discrepancy is due to the heterogeneous nature of GBM and that GFAP may be preferentially expressed in certain GBM subtypes or even subtypes of GBCSCs. Nevertheless, both studies indicate the migration of GBM cells outside of the CNS, and this is unexpected given that clinically recognizable hematogenous metastasis from GBM is extremely rare (211). While the relationship between GFAP and GBM metastasis clearly requires further elucidation, GFAP staining is considered a standard diagnostic marker for GBM for samples taken within the CNS (208, 212–214).

### CD133

CD133, also known as prominin-1, is a protein found on plasma membrane projections and is one of the cluster of differentiation (CD) antigens (215). CD133 is expressed on HSCs (216) and was also found on NSCs in 2003 (20). Singh et al. (20) have identified stem-like cells lacking the expression of neural differentiation markers in pediatric brain tumors that express CD133, and showed that CD133^+^ human GBM cells can initiate tumor formation in the brains of immunodeficient mice (21). Interestingly, these cells also express nestin, indicating the possibility of CD133 expression on progenitor cells. CD133 expression has since been implicated in other cancers, including prostate and colorectal cancer, and an increased proportion of CD133^+^ cells in a tumor correlates with poorer survival (30, 47, 217). GBM tumors that have recurred after radiotherapy or chemotherapy contain an increased percentage of cells that are CD133^+^ compared with the original tumor, presumably due to increased progenitor cell activation (42). Similarly, the CD133^+^ gene transcription signal can distinguish GBM from low-grade tumors and its expression has been attributed to the aggressiveness of the tumor (218). These suggest a key role for CD133 in tumor recurrence and invasion.

However, not all stem cells express CD133. Subsequent studies have shown that tumors grow successfully from CD133^−^ stem-like cells in xenograft models (74, 217, 219) and so identification of CSC cannot be solely based on CD133 expression. As CD133 is not essential for tumor formation, this implies that it is not present on all ESCs. Therefore, CD133 cannot be considered an ESC marker, but is further along on the stem cell hierarchy and can be considered as a marker for progenitor cells.

### CD44

CD44 is a transmembrane glycoprotein and the receptor for the glycosaminoglycan hyaluronan (HA) (220, 221). It is found in a variety of tissues and is expressed on embryonic epithelia during development (222). Multiple isoforms of CD44 exist, altered through splicing and post-translational modifications. CD44s is the most common isoform, but other variants (CD44v) also exist (223). The potential for hundreds of variations on the CD44 receptor may contribute to its involvements in various pathways, including lymphocyte activation, angiogenesis, cytokine release, and cellular adhesion (222). Additionally, CD44 has been implicated in colorectal (224, 225), prostate (67, 226, 227), breast (64, 228–230), head and neck (65, 231), and non-small-cell lung (232) cancers. CD44^+^ cells can generate new tumors similar to the original tumor when xenografted onto mice, but CD44^−^ cells cannot achieve this. (65). These findings are consistent with those indicating that CD44^+^ cells from prostate cancer are more proliferative than CD44^−^ cells and that they also possess some progenitor cell properties (67).

CD44 activates NANOG in breast and ovarian cancers (145) demonstrating a role for CD44 in the regulation of ESCs, supported by the finding that in cancer CD44^+^ cells also express Bmi1 (65). Moreover, tumor aggressiveness and growth can be inhibited by preventing the HA-CD44 interaction (233). This observation suggests a key role for CD44 and its ligand in the development of cancer, although opposing views exist.

Increased CD44 expression is associated with a better outcome in thyroid cancer (234). Similar findings have also been demonstrated in ovarian cancer (235), non-small-cell lung cancer (236), and soft tissue sarcomas (237) although high expression of CD44 is also correlated with increased risks of recurrence (237). This variation is likely due to the CD44 isoform examined, as each study uses a different variant or epitope or CD44 as a whole. Caution is needed when interpreting the data in the attempt to elucidate the precise role of CD44 in cancer.

Li et al. (238) show that CD44 variants are not expressed by GBM, but only in metastases originating from the brain. However, a subsequent study shows that CD44 variants are expressed in 100% of all GBM cell lines and tumors (239). This latter finding has been supported by a more comprehensive study using immunohistochemical staining that demonstrates cells from GBM express CD44s and several other variants of CD44 (240). Furthermore, inhibition of CD44 prevents progression of GBM, indicating a definite role in tumorigenesis (241). However, different GBM cell lines have varying expression of CD44 (242). These results show changeable expression of CD44 in cancer, with some studies finding high expression while others show low expression in the same cancer type (67), indicating CD44 is not essential for tumor formation. Consistent with this observation is the current hypothesis that CD44 is a progenitor cell marker as opposed to an ESC marker. An extensive study of the expression of CD44 in mouse cerebellum show this cell surface marker to be co-expressed with nestin, SOX2, astrocyte specific glutamate transporter and brain lipid binding protein (BLBP), all of which are specific to neural stem/progenitor cells (243). CD44 is also co-expressed with the oligodendrocyte progenitor marker Olig2. This evidence would infer that CD44 is a progenitor cell marker, as it is present on partially differentiated cells.

## Discussion

While there is growing evidence supporting the CSC model of cancer, the field of CSCs in glial tumors remains relatively understudied, as evidenced by the difficulties in identification and characterization of this primitive population. In this article, we review a number of markers published in the recent literature and evaluated their usefulness in CSC research, in the context of GBM. While some markers are key to the identification of CSCs, others have a less defined association, requiring more study to define their precise role in carcinogenesis. It is becoming increasingly apparent that the hierarchical system observed in normal stem cells, such as HSCs, and in other forms of cancer, also applies to GBM.

Cells in GBM express OCT-4 (104, 105), SOX2 (1, 104, 114), pSTAT3 (81, 134), NANOG (104, 152), SALL4 (80, 99), c-Myc (78, 168, 172), KLF4 (58, 190), nestin (20, 21), CD44 (239, 240), CD133 (21, 218), and GFAP (207, 208), highlighting an overlapping hierarchical and heterogeneous population of stem and progenitor cells within GBM. This paper attempts to categorize each of the markers into one of two categories, (1) ESC markers and (2) progenitor cell markers, based on current evidence. NANOG, SALL4, OCT-4, KLF4, SOX2, and pSTAT3 all have essential roles in embryonic development, indicating that they must be expressed on more primitive cells. However, SOX2 and pSTAT3 are also expressed in cells that are more differentiated than ESCs, indicating that expression of a particular marker is not restricted to one cell type or developmental stage.

Current evidence suggests that GFAP, nestin, CD44, and CD133 are found further down in the stem cell hierarchy as they are expressed on more differentiated cells. Although involved in the modulation of tumor aggressiveness, both CD133 (74, 217, 219) and CD44 (67, 243) do not appear to be essential for cancer formation, leading to the inference that these markers represent markers of more differentiated progenitor cells in the hierarchy although they may be co-expressed with nestin and GFAP in “higher up” progenitors. The presence of the same markers on multiple cell types highlights the importance of using multiple markers to properly define and distinguish the most primitive CSCs from their specific but varying downstream lineage and progenitor cells. Such models have already been proposed for non-cancerous stem cells (Figure 4) (24); and in the case of HSCs, a number of markers have been assigned to specific stages of blood cell development (244). A similar paradigm for markers for GBM is currently missing from the literature and establishing a properly defined model of the hierarchy will improve the understanding CSCs in GBM. Additionally, it is important to remember that the stages of stem cell development and maturation are likely not static or strictly defined, but more of a free flowing continuum upon which multiple variations of stem cells can be found. This is particularly important given the high degree of intra- and inter-tumor cellular heterogeneity that is already known to exist within GBM.

Although it has been shown that stem cell markers such as CD133 and CD44 persist on genetically diverse clones (53), the presence of more primitive markers, such as OCT-4, SALL4, NANOG, SOX2, c-Myc, KLF4, and pSTAT3, on different GBM or GBCSC subtypes has not been defined. The ability to identify these extremely primitive CSCs may be key to developing novel and effective treatments for GBM. Finding markers that are consistently expressed by CSC populations within different GBM subtypes may enable effective targeting of CSCs by destroying the “roots” of the cancer. Properly defining the CSC markers will underscore precise identification and characterization of the CSC population in GBM.

## Author Contributions

TI and STT initiated and conceptualized the review. AB drafted the manuscript. AW, ST, LP, PD, and TI critically examined and commented on the manuscript. All authors revised and approved the manuscript.

## Conflict of Interest Statement

The authors declare that the research was conducted in the absence of any commercial or financial relationships that could be construed as a potential conflict of interest.
